# O.3.1-6 Work-related well-being of sport professional at public sector

**DOI:** 10.1093/eurpub/ckad133.143

**Published:** 2023-09-11

**Authors:** Paula Marja Johanna Harmokivi-Saloranta, Mari Punna, Tiina Laiho, Sirpa Laitinen-Väänänen

**Affiliations:** Haaga-Helia Univeristy of Applied Science, Finland; School of Professional Teacher Education JAMK, Finland; paula.harmokivi-saloranta@haaga-helia.fi; Haaga-Helia Univeristy of Applied Science, Finland; School of Professional Teacher Education JAMK, Finland; paula.harmokivi-saloranta@haaga-helia.fi

## Abstract

Sports instructors, who work in small and mid-sized municipalities and the public sector, provide physical activity counselling and group guidance for municipality residents of all age groups. Over the last decade, the work has expanded from group guidance to promote the well-being of residents. Many of them are working independently, having only little collegial or community support. Additionally, only little is known about work-related well-being of their own as well as methods on how to improve it. The aim of this study was to investigate the experiences of a sport professional’s work-related well-being and what factors increase or decrease it.

The study was carried out using qualitative methods. In the study, there were 32 participants who were working as sport instructors in small or mid-size municipalities in Finland. On average, the interviewees were 42 years old and had work experience in the municipality’s sport instructor work for an average of seven years. The data was collected with thematic interviews during the autumn and winter 2022-2023 and it was analyzed using content analysis.

The results showed that work-related well-being was confirmed by the relevance of the work, and by the customers and their success in their own goals. The study also found that the appreciation and sense of community experience also played a significant role in the well-being experienced at work. Work-related well-being is also strengthened by supporting autonomy.

Work-related well-being was weakened by the fragmentation of work and hurry at work. Clients were a strength resource for sport instructors, but they could also increase mental stress. The clients may tell sport instructors about their own illness and loneliness, but workers did not have enough tools to help them. The results also showed that physical problems can affect well-being at work.

The study provides a broader understanding about the art of sports instructors’ work. It makes visible which are the main elements that increase and decrease sport instructors’ work-related well-being. The results can be utilized when implementing activities of sport instructors’ well-being promotion.

The research has been funded by the Finnish Work Environment Fund.

